# Fully Automated Measurement of Cobb Angles in Coronal Plane Spine Radiographs

**DOI:** 10.3390/jcm13144122

**Published:** 2024-07-15

**Authors:** Kenneth Chen, Christoph Stotter, Thomas Klestil, Jennyfer A. Mitterer, Christopher Lepenik, Stefan Nehrer

**Affiliations:** 1 Department for Health Sciences, Medicine and Research, University for Continuing Education Krems, 3500 Krems, Austria; kenneth.chen@donau-uni.ac.at (K.C.); christoph.stotter@moedling.lknoe.at (C.S.); thomas.klestil@moedling.lknoe.at (T.K.); c.lepenik@imagebiopsy.com (C.L.); 2 Department for Orthopedics and Traumatology, Landesklinikum Waidhofen/Ybbs, 3340 Waidhofen an der Ybbs, Austria; 3 Department for Orthopedics and Traumatology, Landesklinikum Baden-Mödling, 2340 Mödling, Austria; 4 Michael-Ogon Laboratory for Orthopaedic Research, Orthopaedic Hospital Vienna-Speising, 1130 Vienna, Austria; jennyferangel.mitterer@oss.at; 5 II. Department of Pediatric Orthopaedics, Orthopaedic Hospital Vienna-Speising, 1130 Vienna, Austria

**Keywords:** scoliosis, spinal deformity, spinal asymmetry, artificial intelligence, machine learning, deep learning

## Abstract

**Background/Objectives:** scoliosis is a three-dimensional structural deformity characterized by lateral and rotational curvature of the spine. The current gold-standard method to assess scoliosis is the measurement of lateral curvature of the spine using the Cobb angle in coronal plane radiographs. The interrater variability for Cobb angle measurements reaches up to 10°. The purpose of this study was to describe and assess the performance of a fully automated method for measuring Cobb angles using a commercially available artificial intelligence (AI) model trained on over 17,000 images, and investigate its interrater/intrarater agreement with a reference standard. **Methods:** in total, 196 AP/PA full-spine radiographs were included in this study. A reference standard was established by four radiologists, defined as the median of their Cobb angle measurements. Independently, an AI-based software, IB Lab SQUIRREL (version 1.0), also performed Cobb angle measurements on the same radiographs. **Results:** after comparing the readers’ Cobb angle end vertebrae selection to the AI’s outputs, 194 curvatures were considered valid for performance assessment, displaying an accuracy of 88.58% in end vertebrae selection. The AI’s performance showed very low absolute bias, with a mean difference and standard deviation of differences from the reference standard of 0.16° ± 0.35° in the Cobb angle measurements. The ICC comparing the reference standard and the AI’s measurements was 0.97. **Conclusions:** the AI model demonstrated good results in the determination of end vertebrae and excellent results in automated Cobb angle measurements compared to radiologists and could serve as a reliable tool in clinical practice and research.

## 1. Introduction

Scoliosis is a three-dimensional structural deformity characterized by lateral and rotational curvature of the spine. It can be broadly classified according to etiology as idiopathic, paralytic, or congenital and further according to age as infantile (0–3 years), juvenile (3–10 years), adolescent (10–18 years), and adult (>18 years) [1,2]. There are alternative, non-invasive measurement methods like infrared thermography, rasterstereography, ultrasound imaging, and smartphone measurements [3,4,5,6]. However, the current standard method to assess scoliosis is the measurement of the spine’s lateral curvature using the Cobb angle in coronal plane radiographs. When scoliosis is defined as a Cobb angle >10°, an estimated 2–3% of the US population and 1–4% of adolescents worldwide are affected, females more often than males [7,8,9,10]. The causes of scoliosis depend on its type and are not yet fully understood. Environmental, degenerative, and genetic causes are being discussed [7,8,9,10,11,12,13].

**Figure 1:**
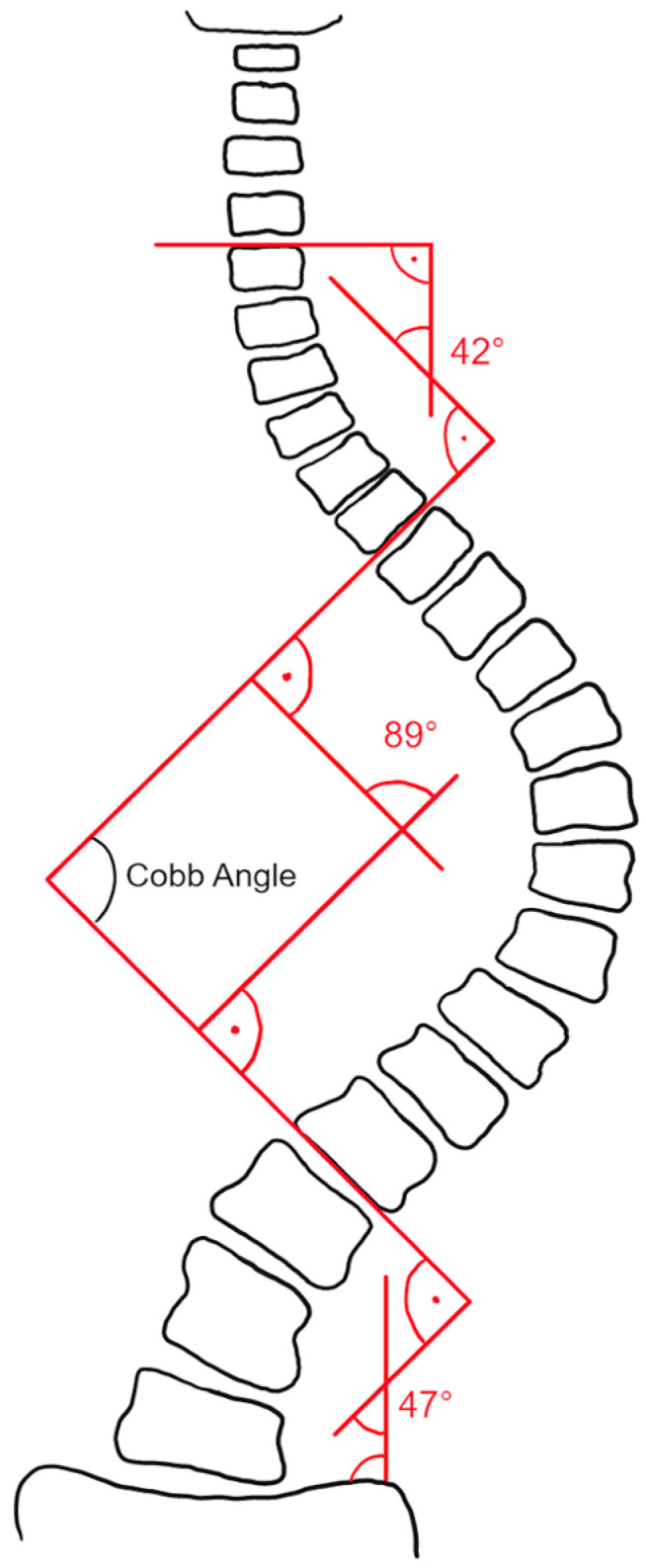
Cobb angle. The angle is measured between a parallel line of the superior endplate of the uppermost vertebrae and the inferior endplate of the lowermost vertebrae (vertebral bodies that are most tilted towards each other) of the scoliotic curves.

The Cobb angle was outlined in 1948 [14] and is the angle formed between a line parallel to the superior endplate of the uppermost vertebra and the inferior endplate of the lowermost vertebra involved in the scoliotic curve [9] (Figure 1). The manual measurement of Cobb angles is a time-consuming task that is prone to error. Several studies have investigated the interrater/intrarater agreement in Cobb angle measurements, with reported variability ranging from 3 to 10°, as well as variability in the definition of the upper and lower end vertebrae [7,15,16,17,18]. As a treatment option, bracing has been shown to reduce the progression of high-risk curves in patients with scoliosis, thereby decreasing the need for surgery [19]. Curves larger than 50° are generally associated with an elevated risk of progression and the need for spinal surgery [20].

Artificial intelligence (AI) in medicine has experienced rapid advances in diagnosis and prognosis and is increasingly being adopted into the orthopedic field [21]. In the context of scoliosis, AI promises great potential to reliably measure the Cobb angle in coronal spine radiographs, showing good to excellent correlation when compared to human raters [7,22,23,24,25,26,27,28]. Earlier studies comparing manual scoliosis measurements to computer-aided methods found higher intrarater agreement for the latter [29]. Recently, AI models with the purpose of predicting curve progression have been introduced, proving higher accuracy than spine surgeons [30,31,32].

In contrast to existing models, IB Lab SQUIRREL was trained on a substantially larger dataset of 17,000 images, surpassing existing models by more than 10-fold [33]. As variations between individual patients need to be represented in a dataset, a deep learning model is highly dependent on the quality and quantity of the input data. A large amount of training data includes a greater amount of variation, ensuring a more robust model. The ground truth established in this study is more elaborate than previous studies and represents high clinical standards [33,34]. The study presented here aims to describe and validate IB Lab SQUIRREL, a commercially available software, and to investigate its interrater/intrarater agreement in measuring Cobb angles in coronal X-rays compared to expert radiologists.

## 2. Materials and Methods

### 2.1. Dataset

In total, 977 anonymized AP/PA full-spine (at least C7 to S1) radiographs of adolescents and adults (10–64 years) from two Austrian clinical sites were acquired. The combined dataset covers various imaging systems and different radiography modalities.

To estimate the number of measurements for standalone performance testing, the method of Lu et al. [35] and reference values from a pilot study were utilized. Based on the inter-expert parameters (µ, σ, and δ) derived from a pilot study, the minimum number of measurements to guarantee sufficient power (90%) for standalone performance testing was found to be 56 curvatures for the Cobb angle measurement. This number was increased substantially to ensure sufficient statistical power.

Prior to sampling, initial quality assurance was performed based on the IB Lab SQUIRREL image requirements, which resulted in the exclusion of 41 images, leaving 936 images (see the “Initial quality assurance” column in Table A1 of Appendix A). Following visual estimation of the main spinal curvature, these images were classified into “mild” (10–20°), “moderate” (20.1–40°), and “severe” (>40°) curvature severity subgroups. A random sample of 67 spines was drawn from each of the spinal curvature severity categories. Because the total number of “severe” spinal curvatures was insufficient (37), an additional 30 images with “moderate” spinal curvatures were sampled from the dataset. This resulted in a final dataset of 201 AP/PA full-spine radiographs, with 67 “mild”, 97 “moderate”, and 37 “severe” curvatures.

### 2.2. Reference Standard

The reference standard was established by four certified radiologists from The Netherlands, with a subspecialty in musculoskeletal radiology. Two of the expert readers had 5–10 years of post-radiology training experience, while the other two readers had 10–15 years of post-radiology training experience. The measurements were performed independently.

The expert readers were also asked to perform quality control on each image prior to performing measurements (see “Reader quality assurance” column of Table A1 in Appendix A). An image was excluded from the study if one or more readers indicated that it did not meet the requirements. This led to the exclusion of a total of five images by the expert readers based on IB Lab SQUIRREL image requirements.

The remaining 196 AP/PA full-spine radiographs originated from 182 patients (27.8 ± 16.9 years (10, 64); 137 female, 59 male). The dataset consists of 102 computed radiographs (CR) and 94 digital radiographs (DX), originating from three different radiography systems, namely Fluorospot Compact FD, syngoMMWP and YSIO X.pree (all Siemens or Siemens Healthineers, Erlangen, Germany). The distribution of the images with respect to the radiography system and modality is shown in Table A2 in Appendix A.

**Figure 2:**
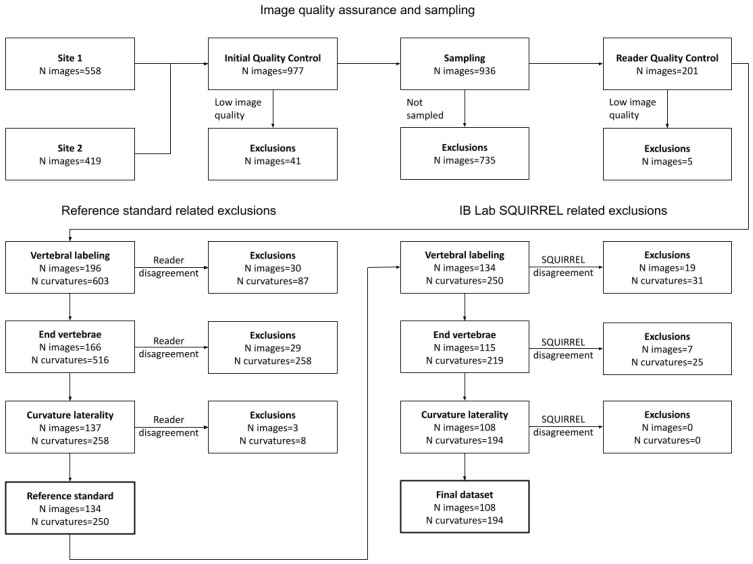
Flowchart depicting exclusions based on the Reference Standard Quality Assurance (QA) process (**left**) and mismatches between the reference standard and IB Lab SQUIRREL (**right**).

When analyzing AP/PA full-spine radiographs, expert reader measurements can differ in various ways, namely vertebral labeling, selection of end vertebrae for Cobb angle measurements, and spinal curvature laterality. Therefore, a multi-stage process was used to ensure a reliable reference standard (see left side of Figure 2):

1. Vertebral labeling from C7 to S1 was compared between the readers. Only images where expert readers agreed on the labeling of vertebrae were used for the study.
2. In order to perform Cobb angle measurements, expert readers needed to define spinal curvatures by choosing appropriate superior/inferior end vertebrae. Small deviations in end vertebrae selection were tolerated; specifically, end vertebrae selection of the readers may extend over three consecutive vertebrae. Curvatures were only included in the study if matching superior/inferior end vertebrae could be determined for all readers.
3. A spinal curvature was only included in the study if the direction of the curve (levo/dextro) matches for all readers.
4. For the remaining curvatures, the reference standard was defined as the median of the readers’ measurements.

To assess IB Lab SQUIRREL’s performance, its outputs were matched to the reference standard (RS) following a similar multi-stage process (see right side of Figure 2):

1. Vertebral labeling from C7 to S1 was compared between the RS and IB Lab SQUIRREL. Only images where IB Lab SQUIRREL agreed with the RS on the labeling of vertebrae were used for the study.
2. A spinal curvature was only included in the study if the end vertebrae of IB Lab SQUIRREL matched one of the superior/inferior end vertebrae of the respective RS end vertebrae. As described above, RS superior/inferior end vertebrae of a curvature were allowed to extend over three consecutive vertebrae. In order to give IB Lab SQUIRREL the same flexibility as the readers regarding end vertebrae selection, IB Lab SQUIRREL’s end vertebrae selections were also allowed to deviate slightly from the RS. However, IB Lab SQUIRREL was only permitted to deviate in such a way that the combination of the RS and IB Lab SQUIRREL superior/inferior end vertebrae of a curvature did not include more than three consecutive vertebrae.
3. A spinal curvature was only included in the study if the direction of the curve (levo/dextro) matched for IB Lab SQUIRREL and RS.

### 2.3. AI Model and Algorithms

**Figure 3:**
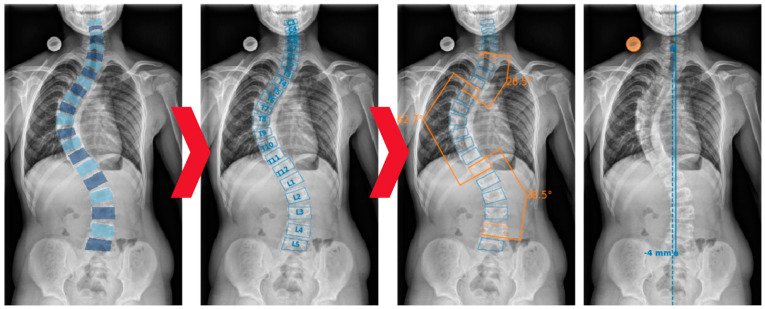
Schematic representation of IB Lab SQUIRREL’s three-step process: (1) Prediction of vertebral bodies, (2) vertebra labeling and (3) determination of Cobb angles and coronal balance.

IB Lab SQUIRREL automates the measurement of Cobb angles and coronal balance on full-spine radiographs through a three-step process: predicting vertebral bodies, labeling the detected vertebrae, and determining the Cobb angles and coronal balance (see Figure 3). This process is supported by machine learning models and advanced pre-/post-processing algorithms.

The first step utilizes high-resolution fully convolutional deep neural networks from the U-Net family [36] to predict segmentation masks and landmark coordinates, identifying vertebral bodies, the sacrum, and essential landmarks. Standard image pre-processing, including resizing, pixel outlier removal, and spectrum normalization, is performed before applying the neural networks.

In the second step, the detected vertebrae are labeled based on the predicted position of the sacrum and specific landmark coordinates.

In the final step, vertebra pairs most tilted towards each other are identified to determine the Cobb angles from their endplates. The coronal balance is derived by measuring the horizontal distance between the center of the C7 vertebra and the sacrum. Although length calibration via a calibration ball is supported by our algorithm, the magnification factor was set to 100% in this study for simplicity.

The deep neural networks were trained on a training dataset of over 17,000 full-spine X-rays annotated with vertebral labels (C7 to S1) using TensorFlow (version 2.5.3) [37]. This independent training dataset was obtained from a third Austrian site and includes a variety of age ranges and scoliosis severity levels, ensuring robustness and generalizability. The dataset was split into three subsets for training, hyperparameter tuning, and performance estimation, ensuring that the scoliosis severity distribution differed by no more than 5% relative to the overall distribution.

Various image augmentation techniques such as random geometric transformations, horizontal flips, and contrast variations were used during training to enhance robustness and generalization.

The model achieved a Dice score of 0.93 for vertebra segmentation and a vertebra classification accuracy of 0.98 on the training test set. While these metrics are significant, the primary goal is the accurate measurement of the Cobb angle.

The final IB Lab SQUIRREL model outputs are internally validated and adjusted by custom algorithms, considering anatomical restrictions and relationships, such as the expected number of vertebrae and their relative locations.

### 2.4. Statistical Analysis

IB Lab SQUIRREL’s performance was assessed in three areas: vertebral labeling, end vertebra selection for Cobb angles, and curvature laterality. The evaluation was based on the percentage of correct labels, end vertebrae, and curvature lateralities, respectively.

For vertebral labeling, accuracy was defined as follows:

Accuracy = (Correct Images)/(All Images) × 100

To be classified as a “Correct Image” in the case of vertebral labeling, all vertebral labels had to match the reference standard (RS).

For Cobb angle end vertebrae selection and curvature laterality, accuracy was defined as follows:

Accuracy = (Correct Curvatures)/(All Curvatures) × 100

To be classified as “Correct Curvature” in the case of Cobb angle end vertebrae selection, both the superior and inferior end vertebrae had to match one of the superior/inferior end vertebrae given in the RS.

For the “Final dataset” (see Figure 2), AI performance was assessed by various statistical methods.

The performance of IB Lab SQUIRREL’s angle and length measurements was assessed for agreement with the RS using Bland–Altman plots. The calculation of confidence intervals for mean difference and limits of agreement was based on Bland and Altman [38].

The accuracy of IB Lab SQUIRREL’s measurements was determined by calculating the mean difference. The precision was measured using the standard deviation of differences, as well as the mean absolute deviation (MAD) and median absolute deviation between the RS and IB Lab SQUIRREL.

Orthogonal linear regression provided insight regarding the presence of an absolute and/or a proportional bias.

To verify the reliability of the expert reads as well as to compare the RS with IB Lab SQUIRREL’s measurements, the reliability coefficient in the form of the intraclass correlation was calculated between the expert readers and between all reads (readers as well as IB Lab SQUIRREL). For this study specifically, we assessed reliability via a 2-way mixed-effects model, single measures, and absolute agreement.

An assessment of interchangeability utilizing the concept from Obuchowski et al. [39] was conducted to show the interchangeability of two modalities, that is, IB Lab SQUIRREL and the assessment of the expert readers. The equivalence index γ was calculated as follows:

γ = *E*(*Y_iT_* − *Y_iRj_*)^2^ − *E*(*Y_iRj_* − *Y_iRj_*_’_)^2^

where *Y_iT_* denotes the result with the new test (*T*) modality, that is, IB Lab SQUIRREL, for image *i*; *Y_iRj_* denotes the result with the existing reference modality (*R*), that is, the expert, by expert *j* for image *i*. An equivalence index γ < 0 provides evidence that IB Lab SQUIRREL is interchangeable with the RS.

The reporting of interrater/intrarater agreement is often lacking a generally accepted standard. To improve comparability, we adhered to the Guidelines of Reporting Reliability and Agreement Studies (GRRAS) [40].

Outlier detection between the RS and IB Lab SQUIRREL measurements was performed using the modified z-score [41], defined for a given measurement *x_i_* as *z_i_* = (*x_i_* − *x*)/(1.4825 × Median Absolute Deviation) with median absolute deviation about the median *x*. Measurements with a modified z-score above 3.5 or below −3.5 were visually inspected to determine the root cause of the deviation.

Data analysis was performed using Python (version 3.8.19) with the scikit-learn (version 1.3.2), scipy (version 1.10.1), statsmodels (version 0.14.1), and pingouin (version 0.5.4) libraries.

## 3. Results

Based on the procedure described in Figure 2, 250 valid curvatures with corresponding Cobb angle measurements remained for the reference standard (RS). IB Lab SQUIRREL provided 572 spinal curvatures with corresponding Cobb angle measurements for 200 AP/PA full-spine radiographs. IB Lab SQUIRREL failed to process one image due to the presence of metalwork. 

Performance was determined by comparing the RS to IB Lab SQUIRREL’s measurements, vertebral labeling, Cobb angle end vertebrae selection, and Cobb angle curvature laterality (see right side of Figure 2). Results are shown in Table 1 below.

Note that vertebral labeling performance is based on a total of 28 images that would have been excluded due to a vertebral labeling mismatch between the reference standard and IB Lab SQUIRREL prior to end vertebrae matching. This means 138 of 166 images would have remained in the image dataset.

After excluding curvatures that did not match between the RS and IB Lab SQUIRREL, 194 curvatures remained for the final dataset. These curvatures originated from 108 AP/PA full-spine radiographs of 101 unique patients (29.3 ± 17.5 years (11–64); 81 female, 27 male). The statistics provided in Table 2 are based on the final dataset. If not explicitly labeled, the statistic is based on the comparison between IB Lab SQUIRREL and the RS.

Additionally, the difference between the IB Lab SQUIRREL Cobb angle measurements and the Cobb angle measurements of the individual most similar reader was assessed. The median of the difference was 0.72° and was smaller than 3° in 90.2% of all cases.

**Figure 4:**
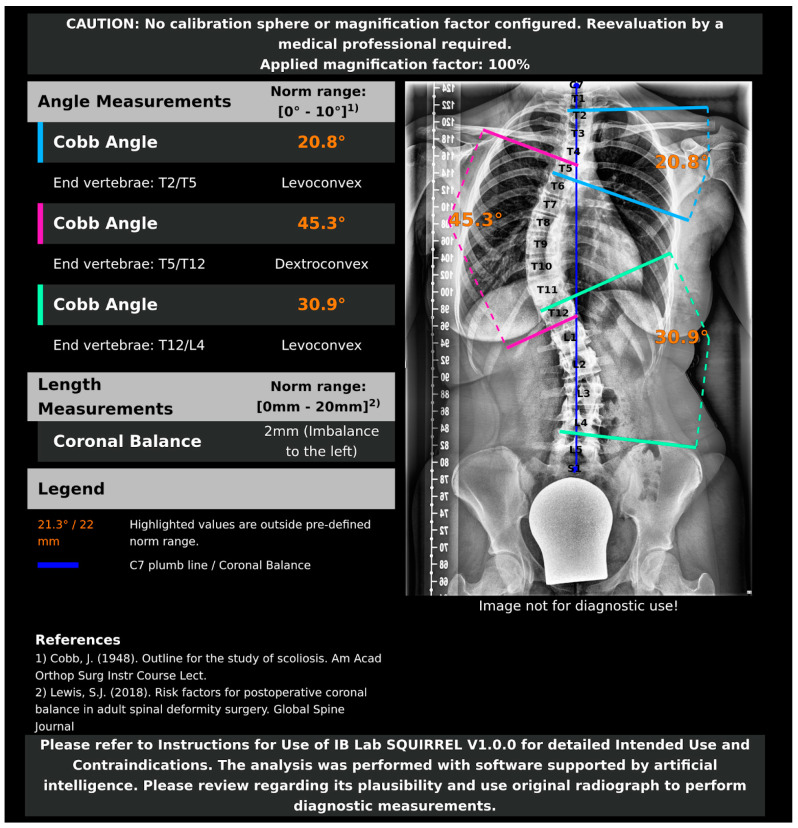
Graphical report, as provided by IB Lab SQUIRREL [14,42].

A graphical report visualizing the measurements by IB Lab SQUIRREL can be found in Figure 4.

### 3.1. Density Plots

**Figure 5:**
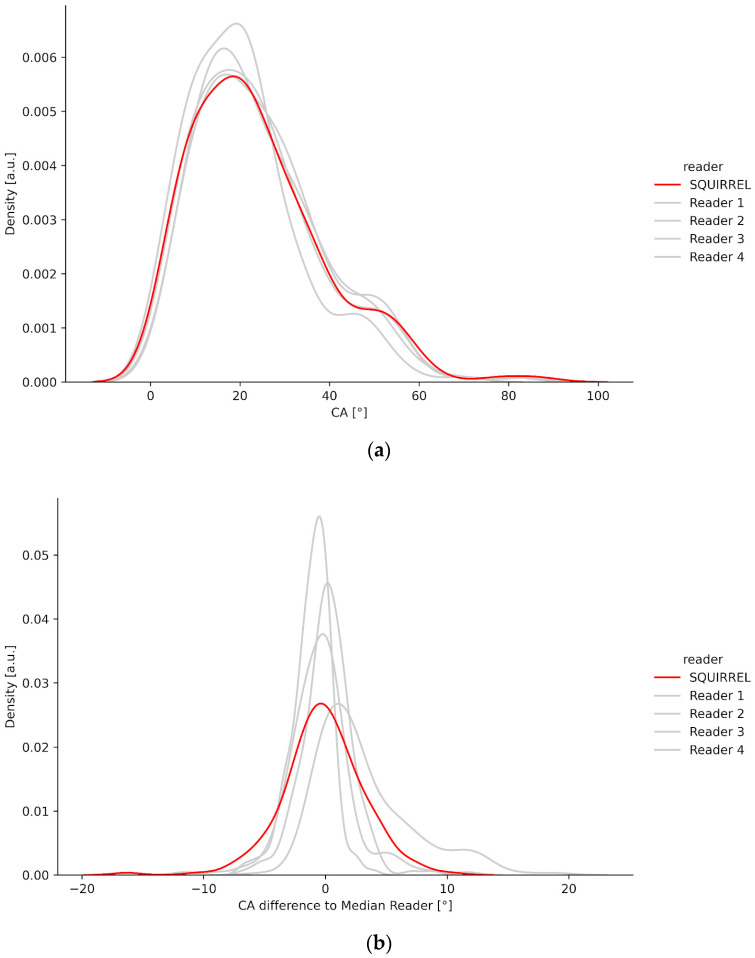
(**a**) Density plot visualizing the distribution of Cobb angle measurements for the four human expert readers and IB Lab SQUIRREL. (**b**) Density plot visualizing the distribution of Cobb angle difference to the median reader.

To visualize IB Lab SQUIRREL’s results in comparison to the human expert readers, we show density plots of the measured Cobb angles as well as the individual differences to the RS (median reader). The probability density function is plotted over the Cobb angle values (Figure 5a) and Cobb angle differences to the RS (Figure 5b), respectively. An analysis of the plots can be found in the discussion.

### 3.2. Bland–Altman and Regression Plots

**Figure 6:**
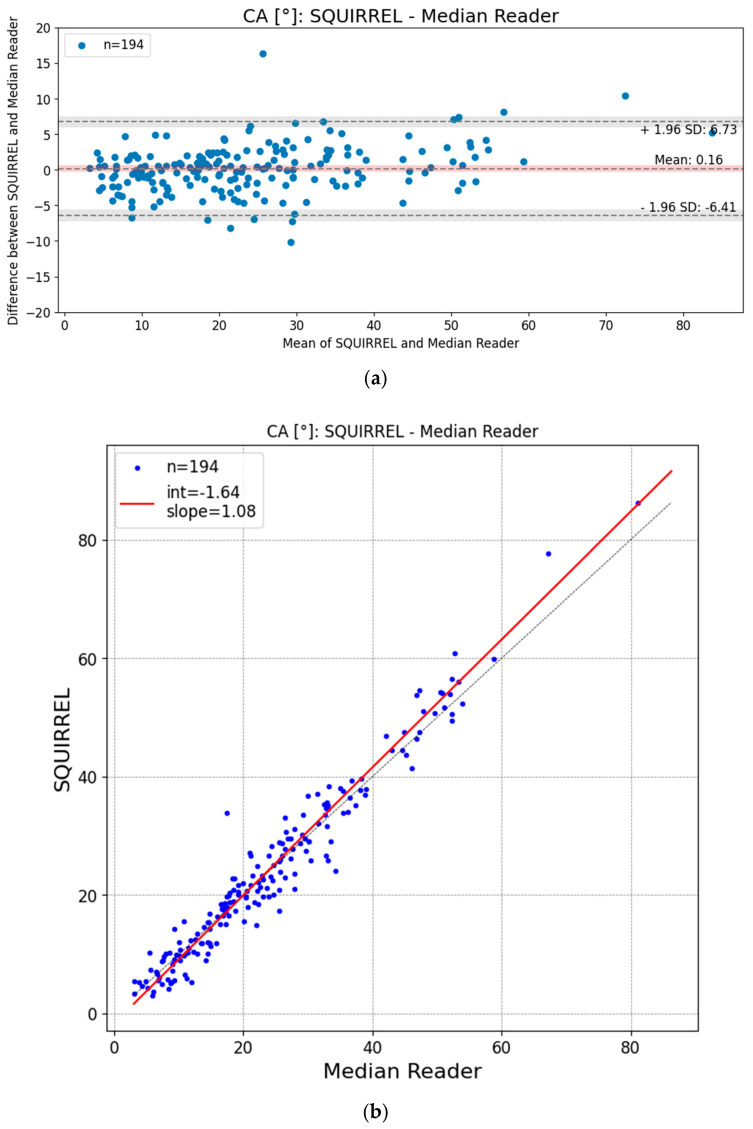
(**a**) Bland–Altman plot with 95% Limits of Agreement (LoA). The red bar indicates the 95% confidence interval of the mean difference between IB Lab SQUIRREL and the reference standard. The gray bars show the 95% confidence interval of the Bland–Altman Limits of Agreement. (**b**) Scatter plot visualizing orthogonal linear regression (OLR, solid line) of IB Lab SQUIRREL and median expert reader Cobb angle (CA) outputs.

In Figure 6a, we display a Bland–Altman plot with 95% Limits of Agreement (LoA), while in Figure 6b, a Regression plot is depicted, visualizing the agreement and correlation between the AI model and the median reader for Cobb angle measurements, respectively.

### 3.3. Intrarater Agreement IB Lab SQUIRREL

Repeating IB Lab SQUIRREL analysis on the same radiograph resulted in an intrarater agreement of 100%.

### 3.4. Outliers

Based on our outlier criterion of z-score >3.5, three Cobb angle measurements were classified as outliers, shown in Table 3 with their respective scores. 

**Figure 7:**
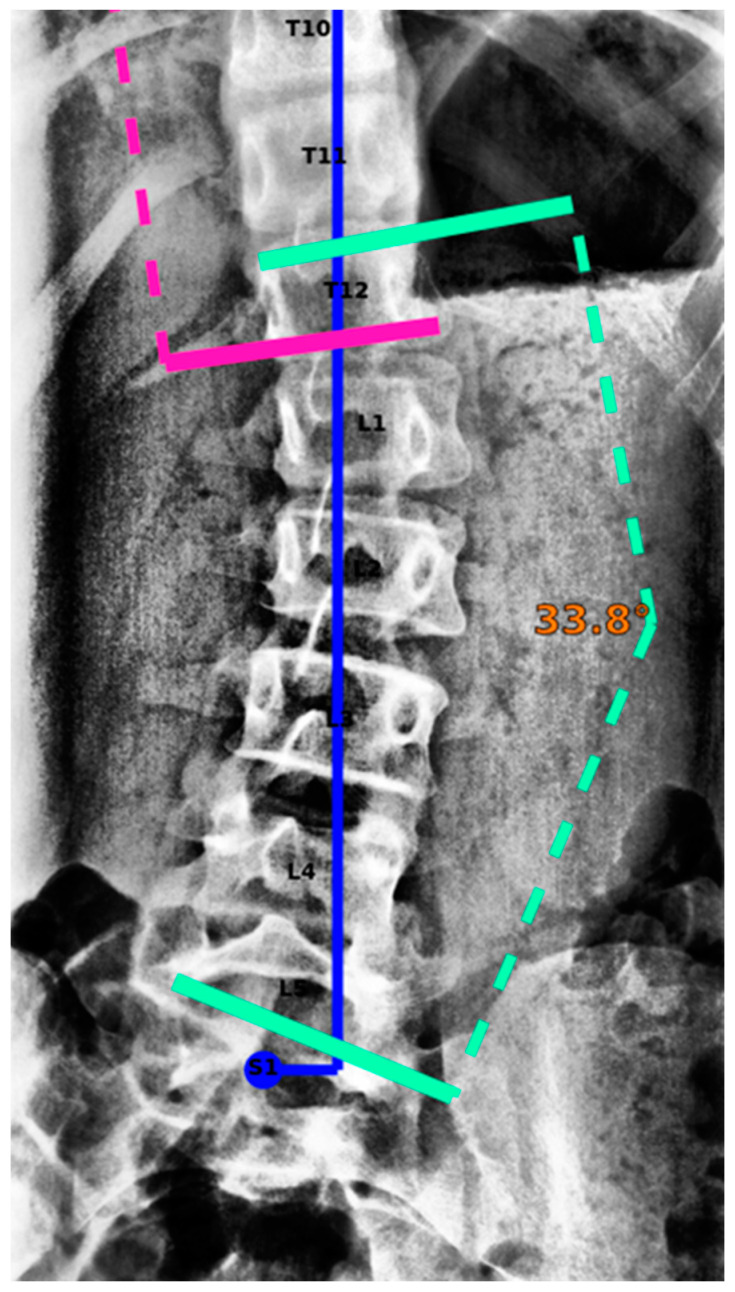
Cobb angle measurement of outlier 1. IB Lab SQUIRREL failed to set the inferior vertebral endplate correctly.

Visual inspection did not reveal any obvious explanation for outliers 1 and 2. The cause of outlier 3, which had the largest z-score of 5.84, could be traced to an error in IB Lab SQUIRREL’s estimation of the lower vertebral endplate (see Figure 7).

Coronal balance results are presented in the Supplementary Materials.

## 4. Discussion

The main finding of this study is that AI, specifically IB Lab SQUIRREL, can accurately identify anatomical landmarks and measure Cobb angles, quantifying scoliosis effectively.

The measurement of Cobb angles in coronal spine radiographs is the gold standard for scoliosis assessment, though it is time-consuming and subject to high rates of interrater/intrarater variability [15,17,19,43]. Previous studies have shown promising results using AI for automated Cobb angle measurements on AP radiographs. These studies have reported ICC values associated with good to excellent agreement, with mean absolute errors ranging from 1° to 8° [7,22,23,24,25,26,27,28]. Comparisons, however, are challenging, due to a lack of external validation, varying statistical quality, and significant differences in the quality and size of the datasets used for training and validation. Unlike earlier research, our study is externally validated and features a substantially larger training dataset, with over 17,000 images. Previous studies utilized training datasets ranging from 1000 to 1500 images [7,22,23], and some even fewer than 500 [24,25,26,27,28]. For instance, Ha et al. utilized a smaller dataset of 1500 images and reported a mean difference of 7.34°, but their study lacked external validation [7]. Similarly, Liu et al. used a dataset of fewer than 200 images and achieved very good agreement, but their training and testing were conducted on data from the same institution [24].

The increased size and diversity of our training dataset offer the promise of a more robust performance, as the AI model is strongly influenced by the quality and variability of the task-specific training data. A larger dataset provides distinct advantages; specifically, the diverse manifestation of the same pathology across different patients might challenge an AI model trained on smaller datasets.

When comparing internally and externally validated models, a significant complication arises due to biases. AI models that are not externally validated tend to perform better on paper, as their training and testing data are drawn from the same dataset of the same institution [21]. Moreover, the distribution and modality of the test data of comparable studies might differ significantly, adding another layer of complexity to comparisons. For instance, the study conducted by Berlin et al. exclusively considered EOS images [28].

Our results exhibit excellent agreement [44] with the reference standard, with an absolute bias (mean difference) of 0.16° and a mean absolute deviation of 2.47°, consistent with previous studies [7,22,23,24,25,26,27,28].

The Bland–Altman LoAs and corresponding 95% confidence intervals (−6.41° [−7.22°; −5.59°], 6.73° [5.91°; 7.54°]) were within the expected interrater variability of 10°.

Despite a slight positive proportional bias (OLR slope [95% CI] of 1.08 [1.04; 1.11]), the measurement error remains within 10% for Cobb angles below 100°.

Interchangeability of IB Lab SQUIRREL with expert readers was demonstrated by a negative equivalence index ɣ [95% CI] of −2.05° [−3.36°; −1.35°]. Thus, the inclusion of the AI model in a group of expert readers is not expected to have a negative effect on agreement within the group.

The ICC indicates excellent reliability for IB Lab SQUIRREL’s Cobb angle measurements, both when compared to the RS (SQUIRREL vs. Median Reader [95% CI]: 0.97 [0.96; 0.98]) and when calculating the ICC between the model and separate expert reader measurements (all reads [95% CI]: 0.94 [0.92; 0.96]).

The density plot of Figure 5a illustrates that the AI model agrees well with the human readers on the full spectrum of Cobb angle values, where the model’s measurements lie between the human readers in the bulk of all Cobb angle values. The plot of Figure 5b illustrates good agreement of the model with the median reader. Note that the comparison between IB Lab SQUIRREL and the human readers is biased as each median reader measurement is based on the measurement of two human readers.

### 4.1. Issues Comparing Human and AI-Based Measurements

As mentioned above, the end vertebrae are defined as the most tilted vertebral endplates of a spinal curve. Although the amount of tilt can be objectively quantified and compared for each vertebral body, 50% of the curvatures in this study had to be excluded because of discrepancies in identifying end vertebrae by the four readers. This is consistent with the literature, as the determination of end vertebrae was identified to be the largest source of error, with interobserver variability ranging from 0.3 to 3.0 levels [15,17]. The decision for strict inclusion criteria, specifically that all readers had to agree on the end vertebrae, was required to ensure a stable RS. IB Lab SQUIRREL showed agreement of 88.58% in end vertebrae determination when compared to the remaining curvatures that were previously agreed on by readers, leading to an additional exclusion of 11% of curvatures.

The current clinical gold standard of scoliosis assessment by using manual measurements demonstrates low interrater/intrarater agreement. Beauchamp et al. reported that the assessment of Cobb angles performed by the same orthopedic surgeons at 8:00 AM and 8:00 PM resulted in an increased Cobb angle measurement by an average of 5° [43]. As we further develop and validate AI models, we should be aware of the limitations of these gold standards and consider strategies for improving them.

The perfect repeatability in AI measurement highlights the potential advantage of automated AI applications over manual reads. However, the AI’s current limitation of not being able to independently assess outliers and suspicious measurements remains a challenge that needs addressing. This is illustrated by the outlier measurement depicted in Figure 7, where the AI model failed to position the line of the inferior endplate correctly. Graphical reports, as available for IB Lab SQUIRREL, can be a valid solution, allowing doctors or researchers to identify nonsensical predictions easily.

### 4.2. Limitations

One major limitation of this study lies in establishing the ground truth for comparisons, as manual measurements can be significantly variable. As shown before, those measurements can vary considerably, with differences of up to 10° for Cobb angle measurements [16]. To resolve this issue, we applied strict exclusion criteria, which in turn involves the risk of potentially introducing bias towards unambiguous cases.

Currently, IB Lab SQUIRREL does not support images with implants/spinal metalwork present. Although initial internal tests have yielded promising results, additional validation is required to assess this capability.

Another notable limitation is that the AI algorithm was trained on data from a single site, potentially impacting its generalizability. However, the present study mitigates this concern by conducting external validation using data from two independent sites, unrelated to the source of the training data. Thus, the reliable applicability of the model is validated across a wide range of images.

## 5. Conclusions

IB Lab SQUIRREL demonstrates excellent and repeatable results in fully automated Cobb angle measurement. It holds promising potential in the field of scoliosis assessment. However, it is important to remember that AI models should be utilized as adjunctive tools that enhance, rather than replace, human spinal deformity assessments.

## Figures and Tables

**Table 1:** Performance of IB Lab SQUIRREL compared to the RS.

Measurement	Accuracy (%)
Vertebral labeling	83.13
End vertebrae	88.58
Curvature laterality	100.00

**Table 2:** Detailed statistics of IB Lab SQUIRREL’s performance in relation to the RS. CI = confidence interval, ICC = intraclass correlation coefficient, OLR = orthogonal linear regression.

Statistic		Result
Mean Difference [95% CI]		0.16° [−0.31°; 0.64°]
Standard Deviation [95% CI]		3.35° [2.86°; 3.87°]
Mean Absolute Deviation [95% CI]		2.47° [2.17°; 2.81°]
Median Absolute Deviation [95% CI]		1.89° [1.58°; 2.21°]
Root Mean Square Error (RMSE) [95% CI]		3.35° [2.86°; 3.87°]
ICC (inter-reader) [95% CI] (Two-way mixed, single measure, agreement)		0.94 [0.89; 0.96]
ICC (all reads) [95% CI] (Two-way mixed, single measure, agreement)		0.94 [0.92; 0.96]
ICC (SQUIRREL vs. Median Reader) [95% CI] (Two-way mixed, single measure, agreement)		0.97 [0.96; 0.98]
Equivalence index ɣ [95% CI] (Interchangeability)		−2.05° [−3.36°; −1.35°]
OLR Intercept [95% CI]		−1.64° [−2.46°; −0.83°]
OLR Slope [95% CI]		1.08 [1.04; 1.11]
Bland–Altman 95% Limits of Agreement (LoA) [95% CI]	Lower:	−6.41° [−7.22°; −5.59°]
	Upper:	6.73° [5.91°; 7.54°]

**Table 3:** Outlier measurements and their z-score.

Outlier ID	Measurement	z-Score
1	Cobb angle	5.84
2	Cobb angle	3.70
3	Cobb angle	−3.81

## Data Availability

Restrictions apply to the availability of these data. Data were obtained from Diagnosezentrum Baden and Diagnostikum Linz and are available from the authors with the permission of Diagnosezentrum Baden and Diagnostikum Linz. Declaration of Generative AI and AI-Assisted Technologies in the Writing Process: During the preparation of this work, the authors used ChatGPT by OpenAI in order to enhance readability and find word synonyms. After using this service, the authors reviewed and edited the content as needed and take full responsibility for the content of the publication.

## Supplementary Materials

The following supporting information can be downloaded at: https://www.mdpi.com/article/10.3390/jcm13144122/s1, Figure S1: Flowchart depicting exclusions based on the Reference standard Quality Assurance (QA) process (left) and mismatches between the reference standard and IB Lab SQUIRREL (right) for Coronal balance (CB). Figure S2: (a) Density plot visualizing the distribution of Coronal balance measurements for the four human expert readers and IB Lab SQUIRREL. (b) Density plot visualizing the distribution of Coronal balance difference to median reader. Figure S3: (a) Bland-Altman plot with 95% Limits of Agreement (LoA). The red bar indicates the 95% confidence interval of the mean difference between IB Lab SQUIRREL and the reference standard. The gray bars show the 95% confidence interval of the Bland-Altman Limits of Agreement. (b) Scatter plot visualizing Orthogonal Linear Regression (OLR, solid line) of IB Lab SQUIRREL and median expert reader Coronal balance outputs. Table S1: Detailed statistics of IB Lab SQUIRREL’s Coronal balance measurement performance in relation to the RS. CI = Confidence Interval, ICC = Intraclass Correlation Coefficient, OLR = Orthogonal Linear Regression. Table S2: Outlier measurements and their z-score.

## Appendix A. Auxiliary Tables

**Table A1:** Image quality assurance. Images were excluded based on IB Lab SQUIRREL’s image requirements.

Quality Assurance Criteria	Initial Quality Assurance (Images Excluded)	Reader Quality Assurance (Images Excluded)	Total
PixelSpacing DICOM header tag missing	12	0	12
Implants/spinal metalwork are present in the image	10	1	11
Bone contours of the vertebrae are not fully visible and/or overlapped (e.g., by calibration devices, radiographic protections, or image artifacts)	6	1	7
Calibration device is not positioned properly	6	0	6
Image is not cropped to the region of interest	4	0	4
Image stitching-related issues (e.g., stitching is not continuous, image stitching artifacts obscure anatomical features, or contrast of stitched images differs too greatly between sub-parts)	3	1	4
Image is of poor radiographic image quality (e.g., noisy images, poor contrast on all or part of the image)	0	1	1
Image is no AP/PA full-spine radiograph	0	0	0
Other (burnt in clinical reads)	0	1	1
Total	41	5	46

**Table A2:** Distribution of manufacturer, model, and modality of the radiography systems of the sampled dataset.

Manufacturer	Model	Modality	Number of Images
Siemens	Fluorospot Compact FD	CR	18
Siemens	Fluorospot Compact FD	DX	71
Siemens	syngoMMWP	CR	84
Siemens Healthineers	YSIO X.pree	DX	23
