# Supplementary material for: Fully Automated Measurement of Cobb Angles in Coronal Plane Spine Radiographs
Source: J Clin Med. 2024 Jul 15;13(14):4122. doi: 10.3390/jcm13144122 (PMC11278017; doi:10.3390/jcm13144122)
Supplement: Supplementary file 1 [file jcm-13-04122-s001.zip › jcm-3024201-supplementary.pdf]

Coronal Balance

Material and Methods

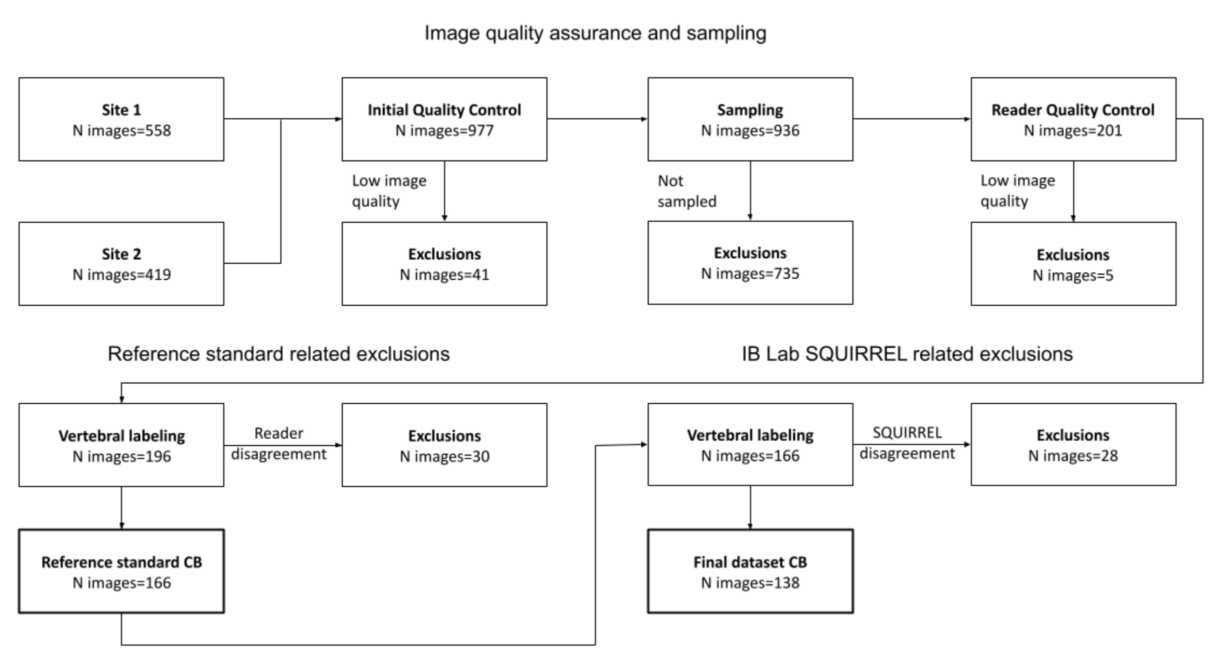

Figure S1: Flowchart depicting exclusions based on the Reference standard Quality Assurance (QA) process (left) and mismatches between the reference standard and IB Lab SQUIRREL (right) for Coronal balance (CB).

Coronal balance was defined as the horizontal distance between the vertebrae centers of C7 and S1 and measured in millimeters.

Following image quality assurance and sampling, a process similar to the one described in Figure 2 of the main document was performed to establish a reliable reference standard (RS) for Coronal balance measurements. The main difference was that for Coronal balance measurements this process was performed on an image rather than curvature level, omitting end vertebrae matching as well as curvature laterality assessment:

1. Vertebral labeling from C7 to S1 was compared between the readers. Only images where expert readers agreed on the labeling of vertebrae were used for the study.
2. For the remaining images, the reference standard was defined as the median of the readers' measurements.

To assess IB Lab SQUIRREL's performance in assessing Coronal balance measurements, its outputs were matched to the RS following a similar process.

1. Vertebral labeling from C7 to S1 was compared between the RS and IB Lab SQUIRREL. Only images where IB Lab SQUIRREL agreed with the RS on the labeling of vertebrae were used for the study.

For the "Final dataset" (see Figure S.1), AI performance of measuring Coronal balance was assessed by comparing the RS and IB Lab SQUIRREL's Coronal balance measurements with the same statistical methods used for the Cobb angle: Bland-Altman plots, mean difference, standard deviation of differences, mean absolute deviation (MAD), median absolute deviation, orthogonal linear regression, ICC (Two-way mixed, single measure, agreement) and the equivalence index γ. To verify the reliability of the expert reads, the reliability coefficient in the form of the intraclass correlation was calculated between the expert readers and between all reads (readers as well as IB Lab SQUIRREL) as well.

Outlier detection between the RS and IB Lab SQUIRREL measurements was performed using the modified z-score.

Results

Based on the procedure described in Figure S.1, 166 images with corresponding Coronal balance measurements remained for the reference standard (RS). IB Lab SQUIRREL provided 200 Coronal balance measurements for 174 AP/PA full-spine radiographs. IB Lab SQUIRREL failed to process one image due to the presence of metalwork and did not provide a Coronal balance measurement when the software failed to label the vertebrae.

After excluding images with vertebral label mismatches between the RS and IB Lab SQUIRREL, 138 AP/PA full-spine radiographs remained for the final dataset. These images originated from 128 unique patients (28.7 ± 17.4 years [11, 64]; 102 female, 36 male). The statistics provided in Table S.1 are based on

the final dataset. If not explicitly labeled, the statistic is based on the comparison between IB Lab SQUIRREL and the RS.

Statistic		Result
Mean Difference [95% CI]		0.55mm [0.28mm; 0.83mm]
Standard Deviation [95% CI]		1.66mm [1.42mm; 1.90mm]
Mean Absolute Deviation [95% CI]		1.38mm [1.21mm; 1.57mm]
Median Absolute Deviation [95% CI]		1.06mm [0.88mm; 1.36mm]
Root Mean Square Error (RMSE) [95% CI]		1.75mm [1.52mm; 1.98mm]
ICC (inter-reader) [95% CI] (Two-way mixed, single measure, agreement)		0.99 [0.99; 0.99]
ICC (all reads)[95% CI] (Two-way mixed, single measure, agreement)		0.99 [0.99; 0.99]
ICC (SQUIRREL vs Median Reader)[95% CI] (Two-way mixed, single measure, agreement)		0.99 [0.99; 1.0]
Equivalence index ɣ [95% CI] (Interchangeability)		-0.74mm [-1.88mm; -0.37mm]
OLR Intercept [95% CI]		0.68mm [0.4mm; 0.95mm]
OLR Slope [95% CI]		0.97 [0.95; 0.99]
Bland-Altman 95% Limits of Agreement (LoA) [95% CI]	Lower:	-2.7mm [-3.18mm; -2.22mm]
	Upper:	3.81mm [3.33mm; 4.29mm]

Table S1: Detailed statistics of IB Lab SQUIRREL's Coronal balance measurement performance in relation to the RS. CI = Confidence Interval, ICC = Intraclass Correlation Coefficient, OLR = Orthogonal Linear Regression

Density Plots

To visualize IB Lab SQUIRREL's results in comparison to the human expert readers, we show density plots of the measured Coronal balance distances as well as the individual differences to the RS (median reader). The probability density function is plotted over the Coronal balance values (Figure S.2a) and Coronal balance differences to the RS (Figure S.2b), respectively. An analysis of the plots can be found in the discussion.

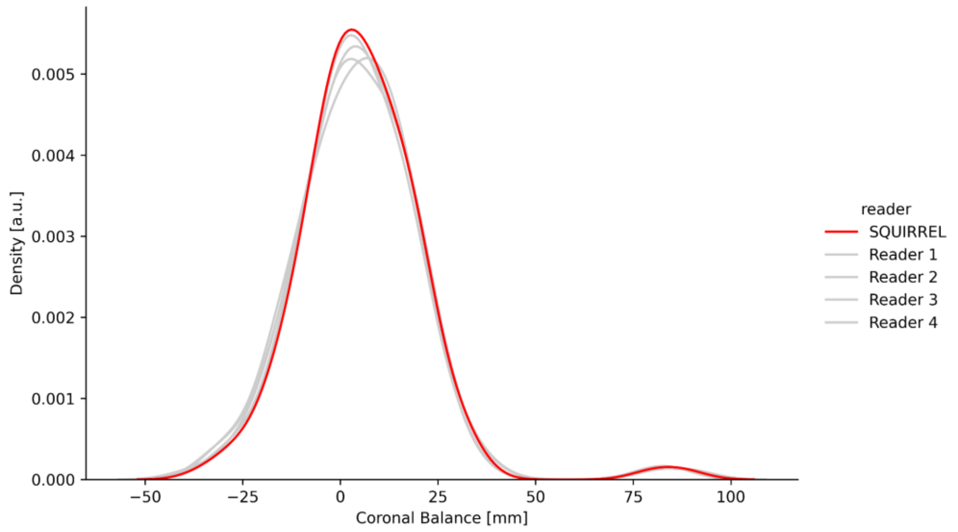

(a)

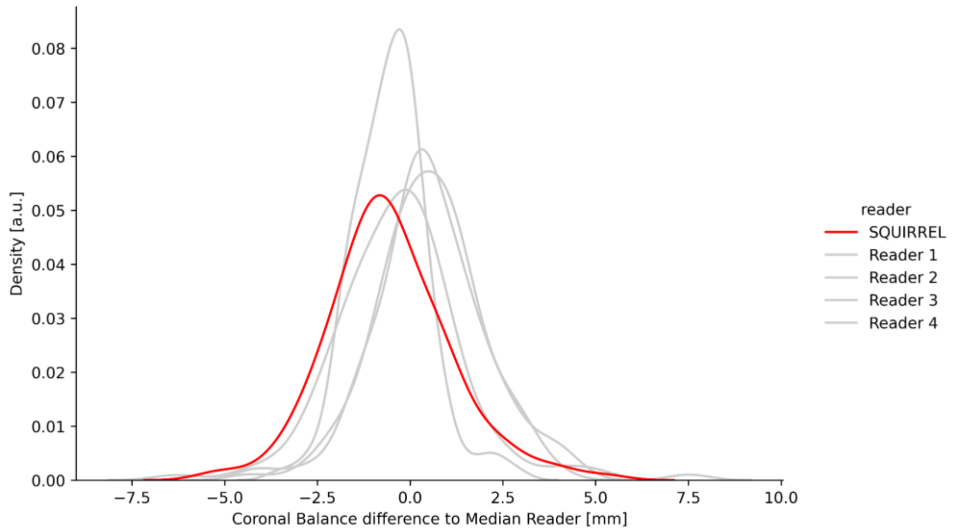

(b)

Figure S2: (a) Density plot visualizing the distribution of Coronal balance measurements for the four human expert readers and IB Lab SQUIRREL. (b) Density plot visualizing the distribution of Coronal balance difference to median reader.

Bland-Altman and Regression Plots

In Figure S.3a, we display a Bland-Altman plot with 95% Limits of Agreement (LoA), while in Figure S.3b a Regression plot is depicted, visualizing the agreement and correlation between the AI model and the median reader for Coronal balance measurements, respectively.

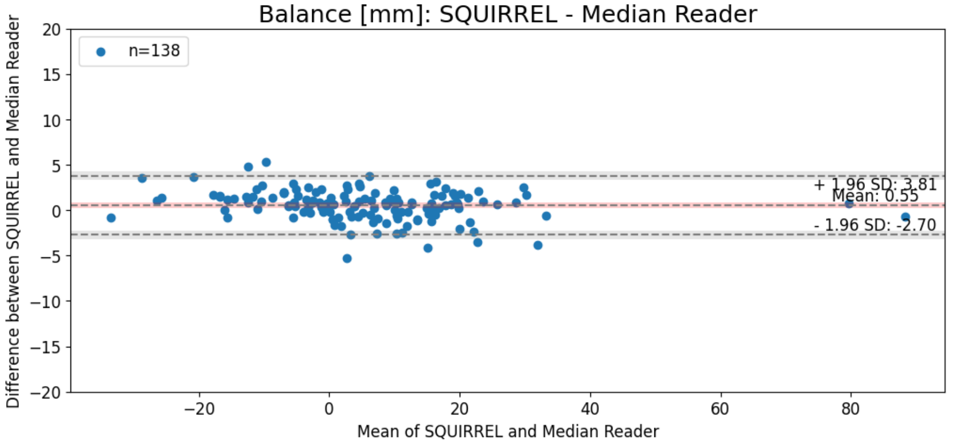

(a)

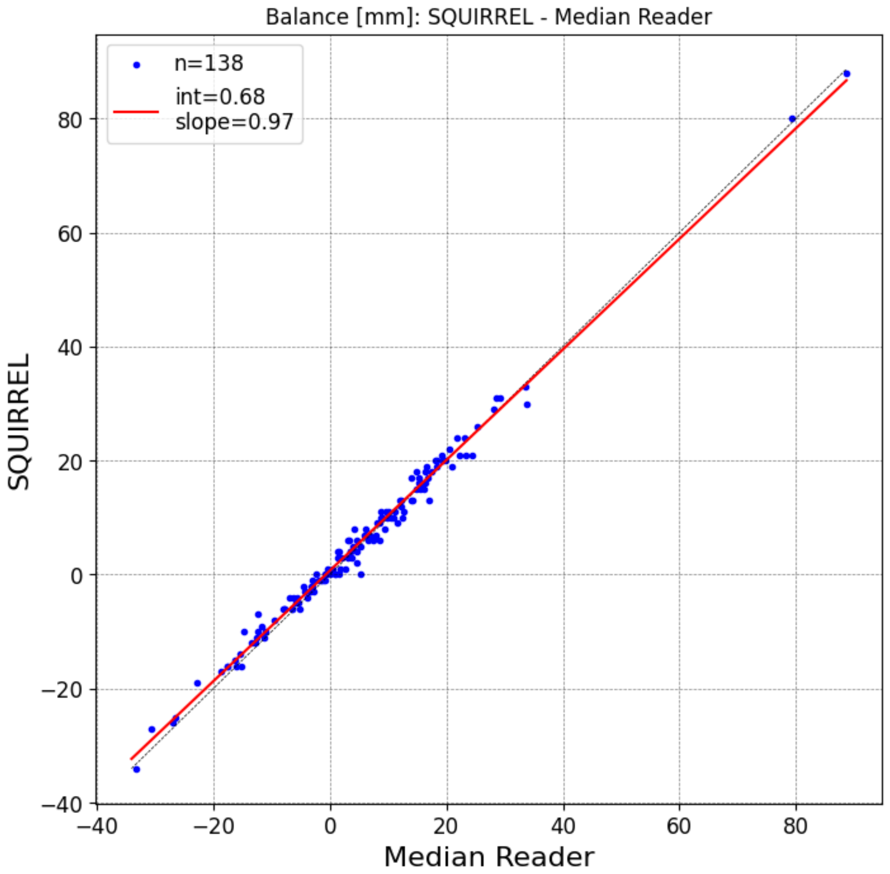

(b)

Figure S3: (a) Bland-Altman plot with 95% Limits of Agreement (LoA). The red bar indicates the 95% confidence interval of the mean difference between IB Lab SQUIRREL and the reference standard. The gray bars show the 95% confidence interval of the Bland-Altman Limits of Agreement. (b) Scatter plot visualizing Orthogonal Linear Regression (OLR, solid line) of IB Lab SQUIRREL and median expert reader Coronal balance outputs.

Outliers

Based on our outlier criterion of z-score >3.5, two Coronal balance measurements were classified as outliers, shown in Table S.2 with their respective scores.

Outlier ID	Measurement	z-Score
4	Coronal balance	-4.37
5	Coronal balance	-3.54

Table S2: Outlier measurements and their z-score.

Visual inspection did not reveal any obvious explanation for outliers 4 and 5.

Discussion

Our results exhibit excellent performance [32] showing very small absolute bias (mean difference) of 0.55mm, and a mean absolute deviation of 1.38mm compared to the reference standard (RS).

Coronal balance measurements of IB Lab SQUIRREL were in agreement with the RS, as Bland-Altman LoAs and corresponding 95% confidence intervals (-2.7mm [-3.18mm; -2.22mm], 3.81mm [3.33mm; 4.29mm]) were within the expected interrater variability of 12mm based on the pilot study.

Coronal balance measurements show a minimal negative proportional bias (OLR slope [95% CI] of 0.97 [0.95; 0.99]).

Interchangeability of IB Lab SQUIRREL with expert readers has been demonstrated by a negative equivalence index ɣ [95% CI] of -0.74mm [-1.88mm; -0.37mm]. Thus, the inclusion of the AI model to a group of expert readers is not expected to have a negative effect on agreement within the group.

The ICC indicates excellent reliability for IB Lab SQUIRREL Coronal balance measurements. This is true both when comparing the AI model's measurements to the RS (SQUIRREL vs. Median Reader [95% CI]: 0.99 [0.99; 1.00]) and when calculating the ICC between the model and separate expert reader measurements (all reads [95% CI]: 0.99 [0.99; 0.99]).

The density plot of Figure S.2a illustrates that the AI model agrees well with the human readers on the full spectrum of Coronal balance values, where the model's measurements lie between the human readers in the bulk of all Coronal balance values. The plot of Figure S.2b illustrates good agreement of the

model with the median reader. Note that the comparison between IB Lab SQUIRREL and the human readers is biased as each median reader measurement is based on the measurement of two human readers.
